# Design, Synthesis, Antiproliferative Actions, and DFT Studies of New Bis–Pyrazoline Derivatives as Dual EGFR/BRAF^V600E^ Inhibitors

**DOI:** 10.3390/ijms24109104

**Published:** 2023-05-22

**Authors:** Lamya H. Al-Wahaibi, Hesham A. Abou-Zied, Eman A. M. Beshr, Bahaa G. M. Youssif, Alaa M. Hayallah, Mohamed Abdel-Aziz

**Affiliations:** 1 Department of Chemistry, College of Sciences, Princess Nourah bint Abdulrahman University, Riyadh 11564, Saudi Arabia; lhalwahaibi@pnu.edu.sa; 2 Medicinal Chemistry Department, Faculty of Pharmacy, Deraya University, Minia 61111, Egypt; drhesham92@yahoo.com (H.A.A.-Z.); abulnil@hotmail.com (M.A.-A.); 3 Medicinal Chemistry Department, Faculty of Pharmacy, Minia University, Minia 61519, Egypt; emanbeshr@yahoo.com; 4 Pharmaceutical Organic Chemistry Department, Faculty of Pharmacy, Assiut University, Assiut 71526, Egypt; 5 Pharmaceutical Organic Chemistry Department, Faculty of Pharmacy, Sphinx University, Assiut 71515, Egypt

**Keywords:** pyrazoline, hybrids, apoptosis, antiproliferative, ADME, DFT

## Abstract

Some new Bis-pyrazoline hybrids **8–17** with dual EGFR and BRAF^V600E^ inhibitors have been developed. The target compounds were synthesized and tested in vitro against four cancer cell lines. Compounds **12**, **15**, and **17** demonstrated strong antiproliferative activity with GI_50_ values of 1.05 µM, 1.50 µM, and 1.20 µM, respectively. Hybrids showed dual inhibition of EGFR and BRAF^V600E^. Compounds **12**, **15**, and **17** inhibited EGFR-like erlotinib and exhibited promising anticancer activity. Compound **12** is the most potent inhibitor of cancer cell proliferation and BRAF^V600E^. Compounds **12** and **17** induced apoptosis by increasing caspase 3, 8, and Bax levels, and resulted in the downregulation of the antiapoptotic Bcl2. The molecular docking studies verified that compounds **12**, **15**, and **17** have the potential to be dual EGFR/BRAF^V600E^ inhibitors. Additionally, in silico ADMET prediction revealed that most synthesized bis-pyrazoline hybrids have low toxicity and adverse effects. DFT studies for the two most active compounds, **12** and **15**, were also carried out. The values of the HOMO and LUMO energies, as well as softness and hardness, were computationally investigated using the DFT method. These findings agreed well with those of the in vitro research and molecular docking study.

## 1. Introduction

**Figure 1:**
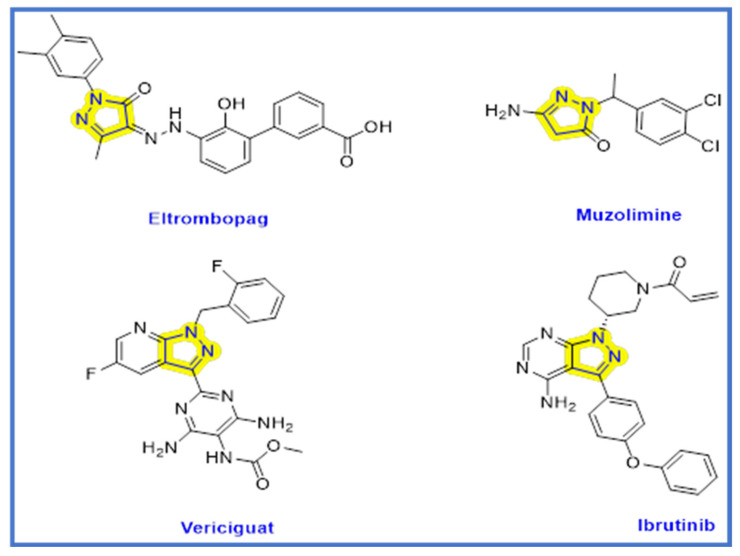
FDA-approved pyrazoline small molecule drugs.

Cancer is one of the major health issues affecting millions of people globally [1,2]. By 2025, the number of newly diagnosed cancer cases globally is anticipated to increase to 19.3 million annually due to changing lifestyles and higher survival rates [3]. Chemotherapy is still the most commonly used cancer treatment today. Although it is beneficial in the treatment of cancer, it has unfavorable side effects due to its non-specific activity on both normal and cancer cells [4]. Therefore, using a small molecule anticancer agent as just a targeted treatment approach is a subject of significant research [4,5]. As of December 2020, 89 small molecule antitumor medications had received approval from both the Chinese National Medical Products Administration (NMPA) and the US Food and Drug Administration (FDA) [4]. Numerous cellular mechanisms and pathways have an impact on the development and spread of various cancer types. Targeting multiple integrated signaling functions with a single molecule is a great way to create potent and less toxic anticancer chemotherapeutic agents [6]. The epidermal growth factor (ERBB) subclass I of the receptor tyrosine protein kinase (RTK) superfamily includes the following members: epidermal growth factor (EGFR)/ERBB1/human epidermal growth factor receptor (HER1), ERBB2/HER2, ERBB3/HER3, and ERBB4/HER4 [7]. The epidermal growth factor receptor (EGFR) is being connected to the development and growth of tumors, as well as to angiogenesis, metastasis, and impaired apoptosis [8]. BRAF (v-raf murine sarcoma viral oncogene homolog B1) is a serine–threonine kinase and mitogen-activated protein kinase (MAPK) signaling cascade activator that is regulated by a GTP-binding protein called RAS [9]. EGFR (epidermal growth factor receptor) and BRAF (B-Raf proto-oncogene, serine/threonine kinase) are both well-studied kinases that play crucial roles in cancer progression [10]. EGFR is a transmembrane receptor that is overexpressed in many types of cancer and is involved in cell proliferation, migration, invasion, and angiogenesis [11]. EGFR mutations are often found in non-small cell lung cancer, head and neck cancer, and colorectal cancer, among others [12]. BRAF, on the other hand, is a downstream effector of the RAS/RAF/MEK/ERK signaling pathway, which is frequently activated in many cancers [12]. Mutations in BRAF are commonly found in melanoma, thyroid cancer, and colorectal cancer [13]. BRAF inhibitors have shown promising results in the treatment of BRAF-mutated melanoma [14]. Extracellular signals regulate cellular growth, differentiation, and survival. In 2002, BRAF mutations were found in a variety of cancers, highlighting the significance of neoplasia [15]. About 8% of all human cancers have BRAF mutations; these mutations are more common in melanomas (40–70%), thyroid (36–53%), and colorectal (5–22%) carcinomas [16]. Contrarily, there is conflicting evidence regarding the sensitivity of thyroid carcinomas to BRAF inhibition, and it has been hypothesized that BRAF^V600E^ thyroid carcinoma cells are less susceptible to BRAF inhibitors such as vemurafenib, because parallel signaling pathways are activated [17]. Since melanoma cells lacking EGFR expression are sensitive to vemurafenib due to the absence of this feedback activation, and since the ectopic expression of EGFR causes resistance to vemurafenib in melanoma cells, the upregulation of EGFR is a prerequisite for the development of this mechanism of drug resistance [17]. Thus, erlotinib or EGFR kinase inhibitors work in synergism with the inhibition of BRAF [18]. As a result, numerous small molecules that act as dual inhibitors and synergize with one another have been medicinally designed as promising anticancer agents [19]. Dual inhibitors targeting both EGFR and BRAF have the potential to provide greater efficacy and overcome resistance compared to single-target inhibitors. Combining EGFR and BRAF inhibition has been shown to result in synergistic antitumor effects in preclinical studies [20]. Furthermore, dual inhibitors may also help to prevent the development of resistance, which is a common problem with single-target inhibitors [21]. Therefore, the development of dual EGFR/BRAF inhibitors is a promising approach in cancer therapy [22]. The process of apoptosis, also known as “programmed cell death”, involves a number of morphological and biochemical events [23]. Bad, Bax, Bid, BcL-Xs, and Bim are proapoptotic proteins. Bcl-2, Bc-LXl, and Bcl-W are antiapoptotic proteins [24]. The outer mitochondrial membrane has become permeable when the ratio of proapoptotic proteins exceeds that of antiapoptotic ones, which triggers a series of events. When cytochrome c is released, caspase-9 and caspase-3 are both activated. Caspases 9 and 3 then stimulate apoptosis by attacking a number of crucial proteins that the cell needs [25]. The pyrazoline scaffold is a five-membered heterocyclic. Pyrazoline is a dihydro pyrazole derivative with one endocyclic double bond in the ring and two nearby N-atoms. Under basic conditions, such as triethylamine, the pyrazoline ring is cyclized by the Michael addition reaction to cyclize chalcones with hydrazine monohydrate [26,27]. Because of its simplicity in preparation and promising pharmacological and biological activities, particularly anticancer properties [28,29], the pyrazoline ring serves as a crucial scaffold in several heterocycles and pharmacologically active compounds [30,31]. The pyrazoline scaffold has been developed into a number of approved drugs as shown in Figure 1 [32]. One of the pyrazoline ring drugs with superb anticancer activity is ibrutinib (Figure 1), which has a fused pyrazoline ring [32]. Ibrutinib is a tyrosine kinase inhibitor which is used to manage mantle cell lymphoma and chronic lymphocytic leukemia [32]. Eltrombopag is a **thrombopoietin receptor agonist** frequently employed to treat thrombocytopenia or aplastic anemia [33]. As a loop diuretic, Muzolimine has been prescribed [34]. Vericiguat is a soluble guanylate cyclase (sGC) agonist used in patients who have chronic systolic heart failure to lessen hospitalization due to heart failure [35]. Pyrazoline derivatives have been also reported to show antidiabetic [36], antioxidant [37], anticancer [28], anti-inflammatory [30], anticonvulsant [38], antibacterial [39], antidepressant [39], antinociceptive [40], antimalarial [41], and antifungal [42] activity. 

**Figure 2:**
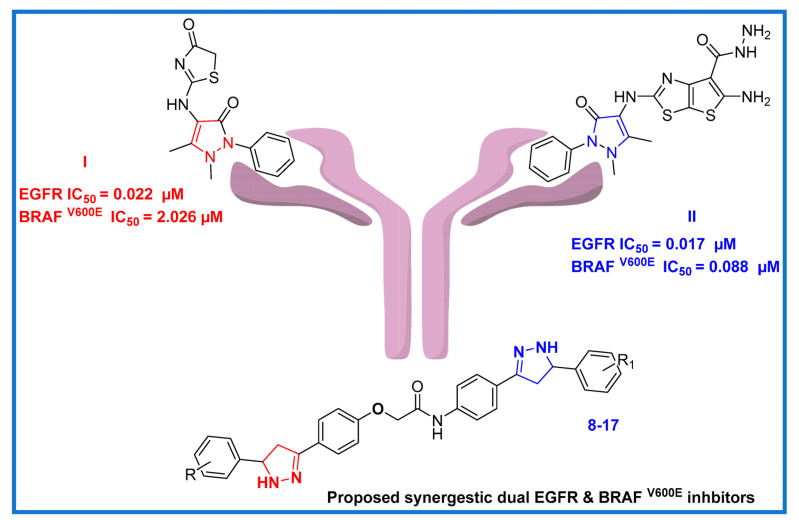
Rational design of recently designed bis-pyrazoline hybrids **8**–**17**.

The year 2022 saw the publication of the novel substituted pyrazoline hybrid **I** (Figure 2), which demonstrated dual antiproliferative activity against EGFR/ BRAF ^V600E^ with IC_50_ values of 0.02 µM and 2.03 µM, respectively, as compared to Sorafenib (IC_50_ = 0.03 µM and 0.04 µM, respectively) [43]. Additionally, another pyrazoline derivative **II** (Figure 2) within the same publication was designed and synthesized as a dual EGFR/ BRAF ^V600E^ inhibitor, and it showed inhibition activity with IC_50_ values of 0.02 µM and 0.09 µM, respectively. 

Molecular hybridization, a promising approach in current drug discovery research, is the combination of two or more pharmacophoric moieties in a single molecular matrix with improved activity and affinity with respect to their parent molecules [44]. By strategically joining one active anticancer drug with another anticancer agent or a carrier molecule, molecular hybridization allows for the design of anticancer medications with improved selectivity, fewer side effects, decreased drug resistance, improved patient compliance, and increased biological potency [44]. 

Our goal is to develop some brand-new, highly effective antiproliferative drugs with minimal side effects and a focus on target specificity. In light of the pyrazoline anticancer studies mentioned above (compound **I** and **II**), we combined two pyrazoline moieties to create compact bis-pyrazoline hybrids **8–17** (Figure 2) and studied their biological antiproliferative activity. Four human cancerous cell lines (breast cancer (MCF-7) cell line, epithelial cancer (A-549) cell line, pancreatic cancer (Panc-1) cell line, and colon cancer (HT-29) cell line) were subjected to an MTT assay to evaluate the newly synthesized bis-pyrazoline hybrids **8–17**. The most effective inhibitory hybrids were tested in vitro for both EGFR and BRAF^V600E^ targets. Interestingly, we found that our novel bis-pyrazoline hybrids (**8–17**) showed higher potency when compared to the previously reported unicyclic pyrazoline derivatives (compounds **I** and **II)**. This increased potency can be attributed to the presence of two pyrazoline rings in our hybrid molecules, which led to enhanced binding affinity and stability at the active site of the enzymes. An ADMET prediction study was calculated to estimate the drug-likeness and toxicity of the novel hybrids. Additionally, molecular docking was conducted to investigate the binding affinities of the novel bis-pyrazoline hybrids within the EGFR and BRAF^V600E^ active sites. The most promising candidates were then put through DFT studies to synergize our in vitro findings with computational quantum mechanics by computing the values of HOMO and LUMO energy, electronegativity, softness, hardness, and potential electrostatic map.

## 2. Results and Discussion

### 2.1. Chemistry

**Scheme 1:**
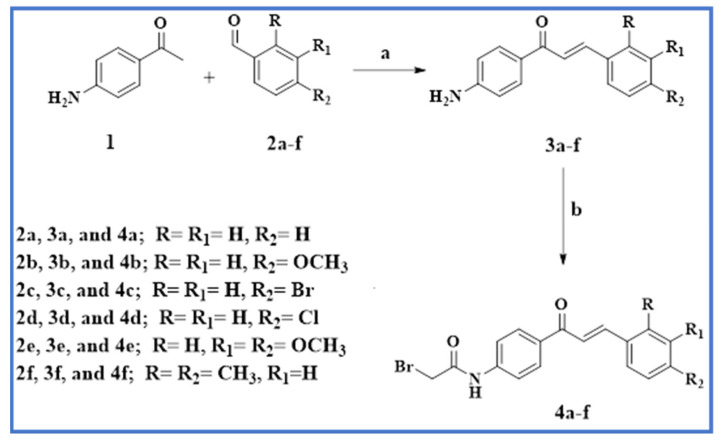
Synthesis of acetylated chalcone derivatives **4a**–**f**.

**Scheme 2:**
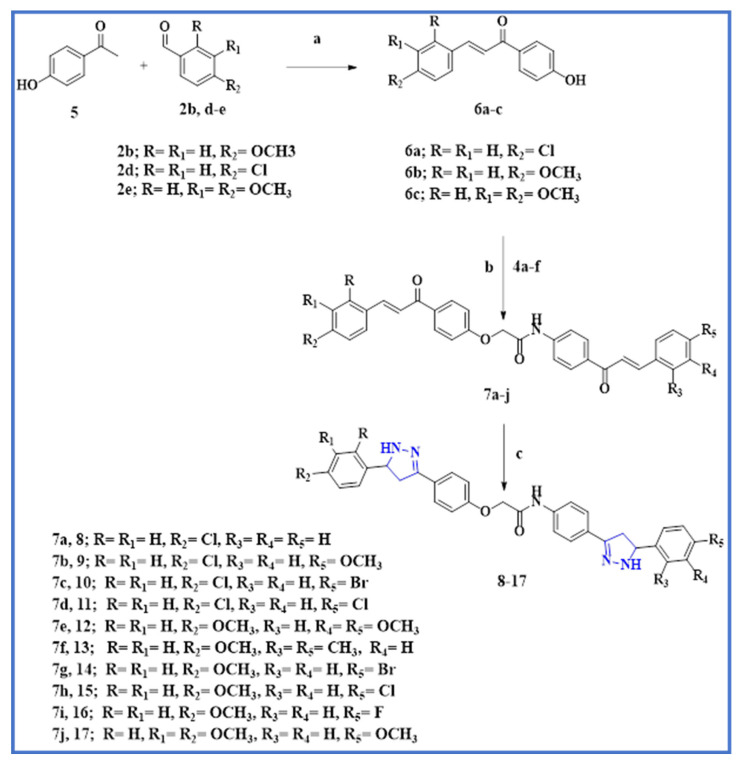
Synthesis of the target compounds **8**–**17**.

Scheme 1 and Scheme 2 describe the synthetic procedures for the intermediates 4a–f and the target bis-pyrazoline novel hybrids **8**–**17**. As shown in Scheme 1, compounds 3a-f were synthesized via base-catalyzed Claisen–Schmidt condensation of 4-aminoacetophenone 1 with substituted benzaldehyde derivatives 2a–f [45]. Chalcones 3a–f were then stirred with bromoacetyl bromide in a potassium carbonate solution in dichloromethane to produce the corresponding acetylated chalcones 4a–f.

Reagents and reaction conditions: (a) NaOH 60%; (b) BrCH_2_COBr/ CH_2_Cl_2_.

4-hydroxy chalcones **6a**–**c** were synthesized using the Claisen–Schmidt reaction from aromatic substituted aldehydes **2b**, **2d**, and **2e**. As shown in Scheme 2, the 4-hydroxy-acetophenone **5** reacts with aldehydes **2b**, **2d**, and **2e** to produce 4-hydroxy-chalcone **6a**–**c**. The bis chalcones **7a**–**j** were synthesized via S_N_2 alkylation. The formation of 4-phenoxy potassium salt from phenolic chalcones **6a**–**c** occurred by reacting them with anhydrous potassium carbonate, which was then alkylated with chalcones **4a**–**f** in refluxing acetone to afford **7a**–**j** in a high yield; see Scheme 2. Finally, these bis chalcones **7a**–**j** were treated with an excess equivalent of hydrazine hydrate to yield novel target bis-pyrazoline hybrids **8**–**17**. 

Reagents and reaction condition: (a) NaOH 60%; (b) K_2_CO_3_, acetone, reflux, 18–20 h; (c) Hydrazine hydrate, absolute ethanol, reflux, 10 h. 

**Figure 3:**
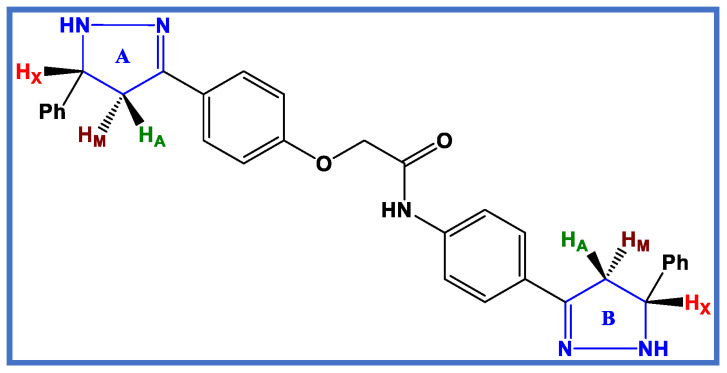
AMX pattern representation of bis pyrazoline target novel hybrids **8**–**17**.

The chemical structures of the bis–pyrazoline target hybrids **8**–**17** were confirmed using spectroscopy, specifically ^1^H NMR and ^13^C NMR. The purity and framework of the synthesized compounds were also confirmed via elemental studies. The ^1^H NMR spectrum of bis pyrazoline **13** as a representative example of bis–pyrazoline hybrids **8**–**17** showed the appearance of an AMX pattern for three protons (H_A_, H_M_, and H_X_) of each pyrazoline ring (ring A and B), which appeared for ring A as a doublet of doublet at δ 2.78 ppm (*J* values of 16.20 and 6.50 Hz) for H_A_, while for the H_A_ proton of ring B it appeared at a field shift at δ 2.66 ppm (*J* values of 16.20 and 6.50 Hz), see Figure 3. However, the splitting of the H_M_ protons of two pyrazoline rings (A and B) caused them to overlap, resulting in a multiplet signal at δ 3.43–3.48 ppm. In a similar way, the H_X_ protons of two pyrazoline rings (A and B) overlapped during their splitting and appeared as a multiplet signal at a higher downfield shift between δ 4.90 and 4.97 ppm (Figure 3). Pyrazoline protons’ transoid and cisoid coupling caused the splitting to be visible. Due to the influence of electronegative nitrogen and the anisotropic effect of the pyrazole ring, the H_X_ proton was more deshielded. At δ 4.74 ppm, the methylene protons (O-CH_2_-CO) of the linker appeared as a singlet signal with two protons. Additionally, a singlet signal that appeared at δ 10.21 ppm was identified as an amide proton NH. 

The ^13^C NMR spectrum of bis pyrazoline **13** revealed chemical shift values at their corresponding positions. The saturated carbons (CH_2_) of pyrazoline Rings A and B were attributed to the peaks at δ 41.25 and 40.50 ppm, respectively, while saturated carbons (CH) showed a more downfield shift at δ 63.59 and 60.75 ppm for pyrazoline Rings A and B, respectively. In addition, a signal at δ 166.90 ppm indicated the presence of amide carbonyl. 

### 2.2. Biological Evaluation

#### 2.2.1. Cell Viability Assay

To test the viability of new substances, the epithelial (MCF-10A) cell line of the normal human mammary gland was used. Compounds **8**–**17** were incubated for four days on MCF-10A cells before being tested for viability using the MTT assay [46,47]. According to Table 1, none of the substances tested were cytotoxic, and cell viability at 50 µM was greater than 87% for the compounds tested.

#### 2.2.2. Antiproliferative Assay

Using the MTT assay and Erlotinib as the reference drug, the antiproliferative role of **8–17** was tested against the four human cancer cell lines MCF-7 (breast cancer cell line), Panc-1 (pancreatic cancer cell line), A-549 (lung cancer cell line), and HT-29 (colon cancer cell line) [48,49,50]. Table 1 shows the median inhibitory concentration (IC_50_) of each tested compound.

Compounds **8**–**17** in general demonstrated promising antiproliferative activity, with the mean IC_50_ (GI_50_) ranging from 1.05 µM to 4.80 µM in comparison to the reference doxorubicin, which had a GI_50_ of 1.10 µM. Compounds **12**, **14**, **15**, and **17** had the highest antiproliferative activity against the four cancer cell lines tested, with GI_50_ values of 1.05, 1.85, 1.50, and 1.20 µM, respectively. It is worth noting that all four compounds contain at least one methoxy group in their structural backbone, indicating the significance of the methoxy group in antiproliferative activity. The tri-methoxy derivative, compound **12** (R = R_1_ = H, R_2 _= OCH_3_, R_3 _= H, R_4 _= R_5_ = OCH_3_), was the most potent derivative, with a GI_50_ value of 1.05 µM, which was equivalent to the reference doxorubicin (GI_50_ = 1.10 µM). Moreover, Table 1 shows that compound **12** was more potent than the reference doxorubicin against the epithelial cancer (A-549) cell line. Compound **17** (R = H, R_1 _= R_2 _= OCH_3_, R_3 _= R_4 _= H, R_5 _= OCH_3_), a tri-methoxy derivative, came second with a GI_50_ value of 1.20 µM, indicating that the number of methoxy groups has a significant effect on the antiproliferative action. Compounds **15** (R = R_1 _= H, R_2 _= OCH_3_, R_3 _= R_4_ = H, R_5 _= Cl) and **14** (R = R_1 _= H, R_2 _= OCH_3_, R_3 _= R_4 _= H, R_5 _= Br) were 1.3-fold and 1.6-fold less potent than compound **17**, with GI_50_ values of 1.50 µM and 1.85 µM, respectively, indicating that halogen atoms are less tolerated than the methoxy group for antiproliferative activity and that chlorine atoms are more favored than bromine ones.

Additionally, we looked into the impact of methoxy group position on antiproliferative activity. Compound **9** (R = R_1_ = H, R_2 _= Cl, R_3 _= R_4 _= H, R_5 _= OCH_3_) was found to be 1.5-fold less potent than compound **15** (R = R_1 _= H, R_2 _= OCH_3_, R_3 _= R_4 _= H, R_5 _= Cl), with a GI_50_ value of 2.3 µM, indicating that the methoxy group was more tolerated at the *para*-position of the phenyl ring which attached to the ring A pyrazoline, rather than at the *para*-position of the phenyl ring which attached to the ring B pyrazoline. 

Compounds **10** (R = R_1 _= H, R_2_ = Cl, R_3_ = R_4_ = H, R_5_ = Br) and **11** (R = R_1_ = H, R_2_ = Cl, R_3_ = R_4_ = H, R_5_ = Cl) showed significant antiproliferative activity, with GI_50_ values of 3.10 µM and 2.9 µM, respectively. This adds to the evidence that methoxy groups were better tolerated than halogen atoms for antiproliferative activity, and that chlorine atoms were more active than bromine ones.

Compound **16** (R = R_1_ = H, R_2_ = OCH_3_, R_3_ = R_4_ = H, R_5_ = F) had a GI_50_ of 3.70 µM and was approximately 2- and 2.5-fold less potent than compounds **14** and **15**, demonstrating the importance of the halogen nature and the order in which activity increased: Cl > Br > F. 

Finally, compounds **8** (R = R_1_ = H, R_2_ = Cl, R_3_ = R_4_ = R_5_ = H) and **13** (R = R_1_ = H, R_2_ = OCH_3_, R_3_ = R_5_ = CH_3_, R_4_ = H) were the least potent derivatives, with GI_50_ values of 4.40 µM and 4.80 µM, respectively. These findings demonstrate that the nature of the substituent at the *para*-position of the phenyl ring which attached to the ring B pyrazoline has a significant effect on antiproliferative activity, which increased in the order OCH_3_ > Cl > Br > F >H > CH_3_.

#### 2.2.3. Structure–Activity Relationship (SAR) Investigation

**Figure 4:**
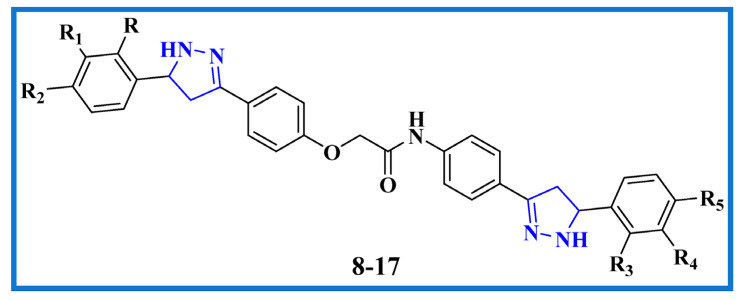
Structure–activity relationship of bis pyrazoline target novel hybrids **8**–**17**.

According to the antiproliferative results, the structure–activity relationship of our new bis-pyrazoline hybrids **8–17** (Figure 4) was as follows:

1. The presence and number of methoxy groups attached to the molecule can significantly impact the antiproliferative activity of the compounds. It is possible that the methoxy group(s) may be contributing to the activity by affecting the interaction of the molecule with its target.
2. The substituent at the phenyl ring attached to the ring B pyrazoline also plays an important role in determining the antiproliferative activity of the compounds. This suggests that the location and nature of the substituent may be affecting the ability to interact with the target and elicit an antiproliferative effect.
3. The activity of the compounds increases in the following order: OCH_3_ > Cl > Br > F > H > CH_3_, indicating that the nature of the substituent plays a critical role in modulating the activity of the compounds.
4. The OCH_3_ group is an electron-donating group, which can increase the electron density of the molecule and enhance its interaction with the target, potentially leading to higher activity. Additionally, the size and shape of the OCH_3_ group may allow it to form favorable interactions with the target, further enhancing its activity.
5. The Cl, Br, and F groups are electron-withdrawing groups, which can decrease the electron density of the molecule and affect its electronic properties and interaction with the target. However, the size and electronegativity of the halogen substituents can also play a role. The larger size of Br and Cl compared to F may allow them to form stronger interactions with the target, potentially leading to higher activity. Additionally, the mild electronegativity of Cl and Br may be better suited for interacting with the target compared to F.
6. The CH_3_ group and H atom are non-polar and relatively small, which may not significantly affect the electronic properties of the molecule or its interaction with the target. However, the size and shape of the substituents can also play a role in steric effects, which can affect the interaction of the molecule with the target. In this case, the CH_3_ group is bulkier than the H group, which may lead to steric hindrance and reduce the ability of the molecule to interact with the target.

Overall, the structure–activity relationship of new bis-pyrazoline hybrids **8**–**17** suggests that the presence and nature of substituents at specific locations on the molecule can significantly impact its antiproliferative activity. This information may be useful in guiding the design and optimization of new bis-pyrazoline hybrids with improved antiproliferative activity.

#### 2.2.4. EGFR Inhibitory Assay

Compounds **12**, **14**, **16**, and **17** were tested for EGFR inhibitory activity as a probable target for antiproliferative activity [43]. Table 2 shows the results as IC_50_ values [51]. 

The results of this test are consistent with the results of the antiproliferative test, in which compounds **12** (R = R_1_ = H, R_2_ = OCH_3_, R_3_ = H, R_4_ = R_5_ = OCH_3_) and **17** (R = R_1_ = H, R_2_ = OCH_3_, R_3_ = H, R_4_ = R_5_ = OCH_3_) were the most effective EGFR inhibitors, with IC_50_ values of 81 ± 5 nM and 87 ± 6 nM, respectively, being equipotent to erlotinib (IC_50_ = 80 ± 5). Compounds **14** (R = R_1_ = H, R_2_ = OCH_3_, R_3_ = R_4_ = H, R_5_ = Br) and **15** (R = R_1_ = H, R_2_ = OCH_3_, R_3_ = R_4_ = H, R_5_ = Cl) demonstrated significant anti-EGFR activity, with IC_50_ values of 104 ± 9 nM and 93 ± 7, respectively, which were 1.3- to 1.15-fold less potent than erlotinib. These results show that compounds **12** and **17** are viable antiproliferative candidates with significant EGFR inhibitory properties.

#### 2.2.5. BRAF^V600E^ Inhibitory Assay

The antiBRAF^V600E^ activity of compounds **12**, **14**, **15**, and **17** was also studied in vitro [45] with erlotinib as a control compound, and the results are shown in Table 2. The findings revealed that the investigated compounds had significant BRAF^V600E^ inhibitory activity, with IC_50_ values ranging from 93 to 125 nM. All of the compounds were less effective than the standard drug erlotinib (IC5_0_ = 60 nM). 

According to Table 2, the most potent EGFR inhibitor, compound **12** (R = R_1_ = H, R_2_ = OCH_3_, R_3_ = H, R_4_ = R_5_ = OCH_3_), was also the most potent BRAF^V600E^ inhibitor, with an IC_50_ value of 93 ± 6 nM. Compounds **15** and **17** were less effective as BRAF^V600E^ inhibitors than erlotinib, with IC_50_ values of 98 ± 8 and 107 ± 9 nM, respectively. Compound **14** demonstrated weak antiBRAF activity with an IC50 value of 125 nM, as shown in Table 2. The results of these in vitro tests revealed that compounds **12** and **17** are promising antiproliferative agents that may act as dual EGFR/BRAF^V600E^ inhibitors.

#### 2.2.6. Apoptotic Marker Activation Assay

##### Caspase 3 Activation Assay

Compounds **12** and **17** were studied for their effects on caspase-3 levels in MCF-7 (breast cancer cell line) and staurosporine as a reference (503.00 ± 4 pg/mL) [46,52,53]. Table 3 summarizes the findings. The results demonstrated that, in comparison to the blank untreated blank cell, derivatives **12** and **17** had significant elevated levels of caspase-3 protein (543.50 ± 4 and 469.50 ± 5 pg/mL, respectively). The most active pyrazoline derivative, **12**, caused caspase-3 protein overexpression (543.50 ± 4 pg/mL) in the MCF-7 cancer cell line, which was 8.5-fold higher than in the untreated blank cell and even higher than staurosporine. The aforementioned results imply that caspase-3 overexpression may be a contributing factor in the antiproliferative effects of the investigated compounds.

##### Caspase-8, Bax, and Bcl-2 Levels Assay

Additionally, the effects of compounds **12** and **17** on the levels of caspase-8, Bax, and Bacl-2 in the MCF-7 cancer cell line and staurosporine as a control were examined [46,52,53]. Table 4 presents the outcomes. 

The results showed that, as compared to staurosporine, the studied compounds considerably raised the levels of Bax and caspase-8. Caspase-8 over-expression was highest in compound **12** (1.87 ng/mL), followed by compound **17** (1.77 ng/mL), and staurosporine (1.80 ng/mL). Furthermore, pyrazoline **12** showed a 37-fold higher induction of Bax (296 pg/mL) than the control untreated MCF-7 cancer cells, whereas staurosporine caused an increase in the Bax level of up to 280 pg/mL. Finally, compound **12** caused an equipotent downregulation of the Bcl-2 protein level (1.05 ng/mL) in comparison to staurosporine (1.10 ng/mL), followed by compound **17** (1.15 ng/mL).

According to the results of the apoptosis assay, compounds **12** and **17** have dual EGFR/BRAF inhibitory properties and show promising antiproliferative apoptotic activity.

### 2.3. In Silico Studies

#### 2.3.1. Docking Study

**Figure 5:**
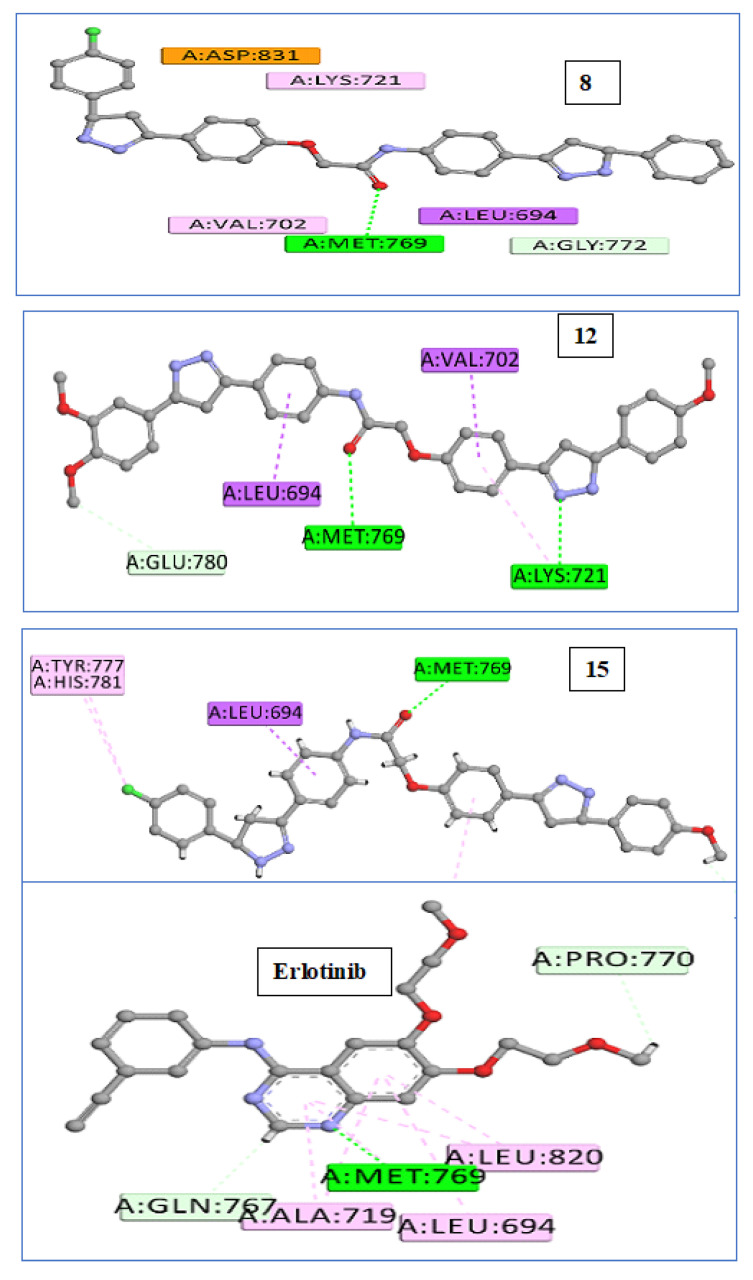
Binding interactions: 2D diagrams of **8**, **12**, **15,** and **erlotinib** within active site of EGFR (PDB ID: 1M17).

**Figure 6:**
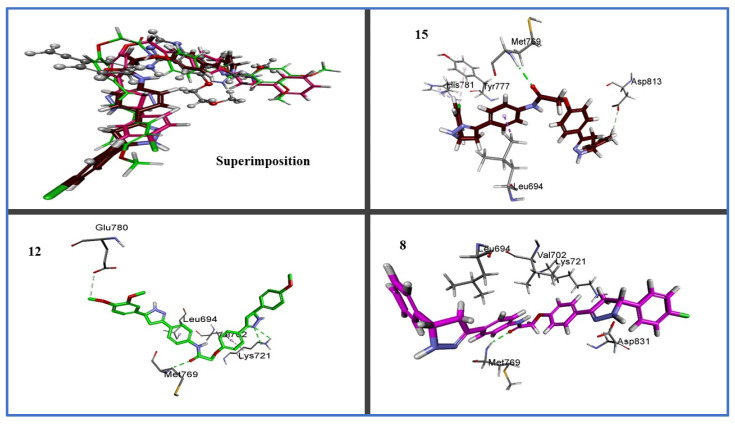
Three-dimensional design of compounds **8**, **12,** and **15** within active site of EGFR (PDB ID: 1M17) showing binding pocket molecular surface occupied completely by novel hybrids.

We used in silico molecular docking simulation models to test compounds **8**, **12**, and **15** against the EGFR and BRAF^V600E^ mutated (BRAFm) [54] proteins to ascertain the binding affinity and mechanisms of inhibition of the most potent compounds (**12**,**15**) and the least potent compound (**8**) against potential cellular targets of this class. By comparing the cellular macromolecules used in this study, the outcomes were very hopeful. Using the Discovery Studio program, molecular docking studies of the crystal structures and binding modes of the EGFR (PDB ID: 1M17) [55] and BRAF^V600E^ (BRAFm; PDB ID: 3OG7) [56] were carried out. The docking model of the co-crystallized ligand (erlotinib) within the active site of the EGFR had a docking score (S) of −7.6 kcal/mol and an RMSD of 1.36 A upon re-docking within the active site of the EGFR. Using this reliable docking model, we investigated how compounds **8**, **12**, and **15** might bind to the EGFR active site. Compound **12** outperforms erlotinib in terms of docking scores (−7.9 kcal/mol). By analyzing the ideal docking pose for hybrids **8**, **12**, and **15**, as displayed in Figure 4, all three of the hybrids were connected to the vital EGFR amino acid Met769 by a regular hydrogen bond acceptor via carbonyl oxygen. Erlotinib’s pyrimidine nitrogen and Met769 exhibit this hydrogen bond as well; see Figure 4. Additionally, compound **12** demonstrated a second hydrogen-binding interaction with LYS 721 in addition to the carbon hydrogen, as well as pi-sigma interactions with the amino acid residues Glu 780, Leu 694, and Val 702 (Figure 5 and Figure 6), which agrees with the EGFR inhibition assay’s in vitro results.

Additionally, due to their similar settling profiles at the EGFR active site, compounds **8**, **12**, and **17** demonstrated perfect crossover with each other and the co-crystallized ligand erlotinib (Figure 5), which is illustrated by the superimposition of the co-crystallized molecule **erlotinib** (gray, ball and stick)**, 8** (pink), **12** (green), and **15** (brown) within EGFR (Figure 5).

**Figure 7:**
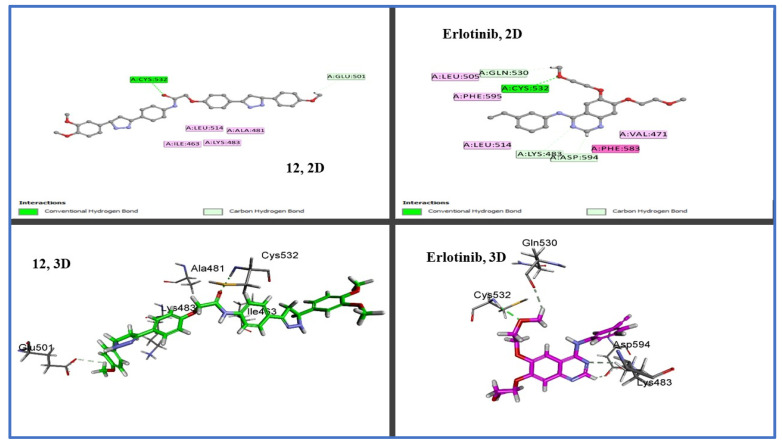
Two-dimensional and three-dimensional binding interaction diagrams of **12** and **erlotinib** within active site of BRAF^V600E^ (BRAFm; PDB ID: 3OG7).

Here, the most active hybrid **12** was docked once more within the active site of BRAFm. It is interesting to note that test hybrid **12** had a similar docking score (−7.2 kcal/mol) to erlotinib (−7.3 kcal/mol). A visual inspection of the 2D drawing of the molecular docking process for compound **12** used in the molecular docking studies, as shown in Figure 7, revealed good fitting within the BRAFm active site with a number of hydrophobic and hydrogen-bonding interactions. Furthermore, interactions between compound **12** and the active site of BRAF revealed one typical H- bond with Cys532, like with erlotinib; see Figure 6.

#### 2.3.2. Density Functional Theory (DFT) Studies

**Figure 8:**
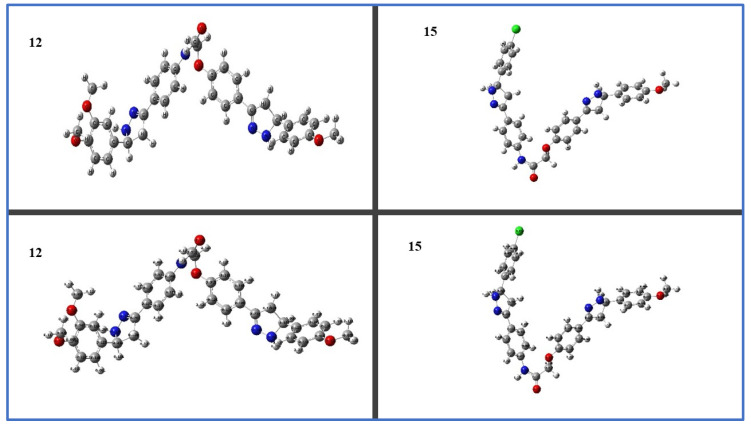
Mullikan charge distribution and optimized geometries for **12** and **15**.

Quantum HOMO and LUMO energy and molecule electrostatic surface potential (MEP) were calculated to better understand the cause of the antiproliferative activity of our target pyrazoline hybrids, and also in order to identify the active groups in the chemical structure of these molecules. Using the DFT/B3LYP level and a basis set of 6-31G (d,p), geometric optimization of the synthesized hybrids **12** and **15** in the ground state was carried out. Figure 8 shows the calculated Mullikan charge distributions and optimized geometries for each atom.

#### 2.3.3. Frontier Molecular Orbitals (FMOs) and Electrostatic Potential of Molecules (MEP)

**Figure 9:**
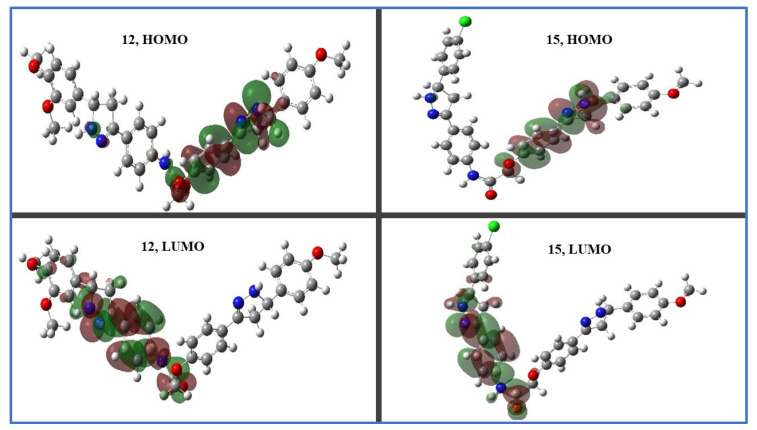
Distribution of molecular orbitals in space of hybrids **12** and **15**.

Frontier molecular orbital (FMO) studies can help us to understand the nature of intramolecular charge separation, electronic excitation, and electron density transition in a molecule [57]. The analysis of E_HOMO_ and E_LUMO_ molecular orbitals, which stand for the highest occupied and lowest unoccupied molecular orbital energies, respectively, is related to the chemical reactivity and stability. Compounds **12** and **15** have HOMO spatial distributions that are primarily distributed on their pyrazoline ring (A) and phenolate moieties (the electron transfer zones), while their LUMO spatial distributions are located on the other pyrazoline ring (B) and aniline moieties (the electron acceptor zones); see Figure 9. 

The electron affinity ***EA*** and ionization potential ***IP***, as well as chemical potential ***μ***, electronegativity ***χ***, hardness ***η***, and softness ***S*** (Table 5), were correlated with the E_HOMO_ and E_LUMO_ [58] which were calculated from the following equations [59]:

***IP = Ionization Potential = −EHOMO,***

***EA = Electron Affinity = −ELUMO,***

***χ = Electronegativity = (IP + EA)/2,***

***ϕ = Chemical potential = −χ***

***η = Chemical hardness = (IP − EA)/2,***

***σ = Chemical softness = 1/(2η)***

The HOMO–LUMO energy gap is linked to the electronic transfer from the ground to the higher energy state which demonstrates how easily the molecule can move an electron from the HOMO level to the LUMO level [60]. The tri-methoxy derivative, compound **12** (R = R_1_ = H, R_2_ = OCH_3_, R_3_ = H, R_4_ = R_5_ = OCH_3_), has lower ΔE (3.962 eV) than the mono-methoxy derivative **15** (R = R_1_ = H, R_2_ = OCH_3_, R_3_ = R_4_ = H, R_5_ = Cl) which revealed ΔE (4.018 eV), demonstrating how the methoxy group is important for reducing the HOMO-LUMO gap and how that relationship directly increases antiproliferative activity. The most advantageous acid–base interaction could occur between hard/hard or soft/soft pairs according to the hard and soft acids and bases (HSABs) principle [61]. The highest hardness ***η*** may refer to a molecule’s stability, whereas softness **S** may refer to a molecule’s effectiveness and reactivity [62]. Additionally, hybrid **12** was softer than hybrid **15**, which enhances its chemical reactivity and reduces its kinetic stability, proving that halogen atoms were less tolerable for the antiproliferative activity than methoxy groups. The ability to withdraw electrons is correlated with electronegativity ***χ***, which measures the resistance to losing electron density. The electronegativity ***χ*** of compound **15** is higher than that of compound **12**. The energy that may be absorbed or released by a change in the particle number is known as the chemical potential ***μ*** in thermodynamics. Because the free energy is reduced, particles frequently move from regions of higher chemical potential to regions of lower chemical potential. Compound **12** has greater chemical potential than compound **15**, which is consistent with the values for electron affinity ***EA*** and hardness ***η***. 

Overall, the DFT studies conducted in this study provide important insights into the electronic properties of the synthesized bis–pyrazoline derivatives. The comparison between compound **12** and compound **15** highlights the crucial role of the methoxy group in reducing the HOMO-LUMO gap, which in turn increases the antiproliferative activity of the compound. The findings also suggest that the presence of halogen atoms in compound **15** may reduce its antiproliferative activity. These insights can help to guide future drug design efforts aimed at developing more potent and effective antiproliferative agents.

**Figure 10:**
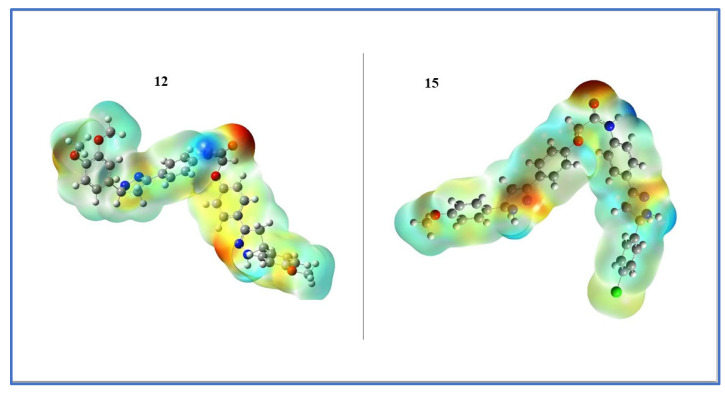
Electrostatic potential maps for **12** and **15** that have been calculated.

The use of molecular electrostatic potential plots can help one to understand how drug-like substances interact with their environment [63]. The optimized structure produces the three-dimensional reactive sites that are electrophilic and nucleophilic. The various shades signify various molecular surface electrostatic potential. In order of decreasing electrostatic potential, the colors are red, orange, yellow, green, and blue [64]. A red color surface indicates an extremely electron-rich (nucleophilic) site, a blue color surface is an electron-deficient (electrophilic) site, and a green color surface reveals an electrically neutral region. It is anticipated that the binding site in the receptor will have opposing electrostatic potential values [65]. Binding interactions are caused by variations in the ligand electrostatic potential on its surface. The molecular electrostatic potential study thus offers details on the ligand strategy and the nature of binding with biological receptors. Figure 10 shows the electrostatic potential surface of the title compounds **12** and **15**. 

The carbonyl oxygen atom of the linker between two pyrazoline rings (A and B) has negative potential (red/yellow color), as seen on the electrostatic surface, which, in the earlier docking modeling, formed a hydrogen acceptor binding interaction with the crucial Met 769 residue of the EGFR receptor active site, while the linker amide hydrogen atom developed a positive potential, and finally the remainder of the molecule was visible in the area of neutral potential. 

To approximate the local intermolecular hardness and softness of the outer molecular regions, one can analyze the electronic contribution to the molecular electrostatic potential. The above information can be used to infer that the linker region has a higher softness value due to the presence of the negatively charged oxygen atom. Conversely, the remainder of the molecule, which is located in the area of neutral potential, is expected to have a higher hardness value. By combining this information with the earlier docking modeling results, it may be possible to gain a better understanding of the molecular interactions that are taking place, and potentially identify areas where modifications could be made to enhance the reactivity of our hybrids.

In conclusion, the DFT studies performed in this study provide valuable insights into the electronic properties of the title compounds and their binding with biological receptors. The findings can be used to guide the design of new compounds with improved antiproliferative activity and binding affinity to biological receptors.

### 2.4. ADMET Evaluation

**Figure 11:**
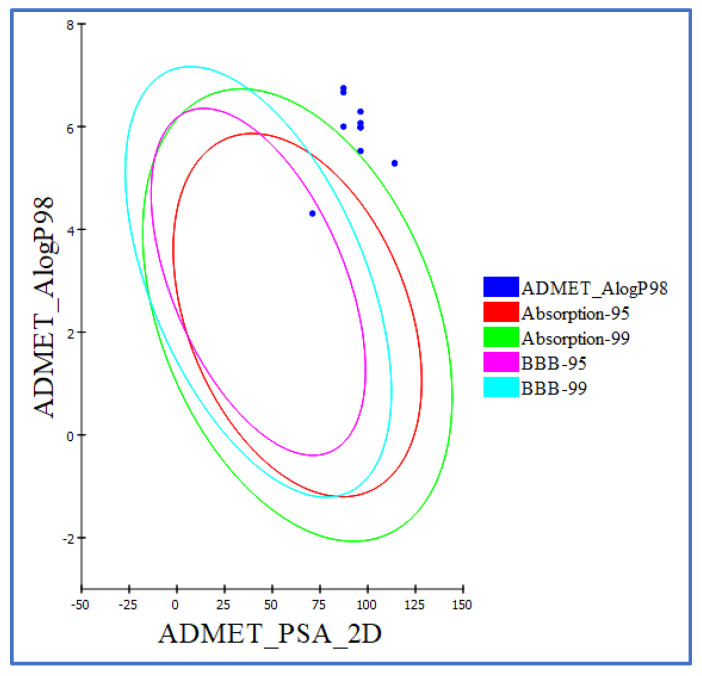
The expected ADMET study.

We conducted additional ADMET studies for the synthesized compounds to gain more knowledge about these crucial activities as a result of the intriguing in vitro and in silico docking/DFT results [66]. Erlotinib was used as the known compound in the ADMET studies. All compound ADMET descriptors were predicted using Discovery Studio 4.0. Table 6 provides a list of the predicted descriptors. All of the hybrids had an estimated poor intestinal absorption (absorption level = 2), making them all promising candidates for the local treatment of GIT tumors. The majority of the novel hybrids displayed an ADME aqueous solubility level of 2 (low aqueous solubility), meaning that the solubility of hybrids is pH-dependent. To enable ionization, solubility rises and pH falls. Making hydrochloride salt is an additional method to increase the solubility of these hybrids. All the novel hybrids in the ADMET plot had a blood–brain barrier (BBB) level of 4, which prevented them from crossing the blood–brain barrier. Drug bioavailability was associated with the essential property 2D polar surface area (ADMET 2D PSA). Using calculated 2D polar surface area (PSA 2D) and atom-based Log P98 (A log P98) properties, the results are displayed as a 2D ADMET plot (Figure 11). Low bioavailability and passive absorption are characteristics of molecules with PSA < 140 [67]. The tested hybrids’ PSA values ranged from 87.31 to 116.01; so, it was expected that they would exhibit passive oral absorption, as seen in Table 6. Using a 2D chemical structure as the input, the cytochrome P450 2D6 (CYP2D6) model forecasts CYP2D6 enzyme inhibition. The majority of drug–drug interaction cases involve the liver enzyme CYP2D6, which is involved in the metabolism of a wide variety of substrates [68]. As a result, an experiment to inhibit CYP2D6 is necessary as part of the regulatory processes used in the drug discovery and development process [69]. All of the examined hybrids were predicted to be CYP2D6 non-inhibitors. The side effect of liver dysfunction is therefore not anticipated after administering these hybrids. The plasma protein binding model foretells whether a substance will be strongly bound (>90% bound) to blood carrier proteins. The majority of hybrids were anticipated to bind plasma protein (>90%); see Table 6. 

### 2.5. In Silico Toxicity Predictions

Using the Discovery Studio software, (V 16. 1. 0. 15350) toxicity prediction was conducted for the synthesized compounds using the constructed and validated models [70]. The FDA’s rodent carcinogenicity test determines the likelihood that a chemical structure will cause cancer in rats. The rat maximum tolerated dose (MTD) was used, which forecasts the rat MTD of a chemical substance [71]. When a chemical compound is tested for toxicity, the rat oral LD50, which forecasts the rat oral acute median lethal dose (LD50), is used [72]. In the Draize test, ocular irritancy determines whether a specific compound is likely to be an ocular irritant and how severe the irritation will be [73]. In a test on rabbits, skin irritancy determines whether a substance is likely to cause skin irritation and how severe it will be. Most compounds demonstrated in silico low toxicity and adverse effects against the tested models, as shown in Table 7. All of the tested hybrids were predicted to be non-carcinogenic, starting with FDA rodent carcinogenicity. The hybrids demonstrated maximum tolerated doses that were 0.11 to 0.21 g/kg body weight higher than erlotinib’s (0.089 g/kg body weight) in the rat maximum tolerated dose model. The tested compounds had oral LD50 values for the rat oral LD50 model that ranged from 0.707 to 10.248 mg/kg body weight/day compared to erlotinib (0.662 mg/kg body weight/day). Additionally, all hybrids were predicted to be mild and non-irritating against models for skin and ocular irritancy, respectively.

In summary, the ADMET studies conducted in this study provide essential information on the potential efficacy, safety, and pharmacokinetics of the novel hybrids. The information gained from these studies is crucial in guiding drug discovery and development and can help in identifying promising drug candidates for further testing and development.

## 3. Materials and Methods

### 3.1. Chemistry

#### 3.1.1. General Details: See Appendix SA (Supplementary File)

Acetylated chalcones **4a**–**f** and 4-hydroxychalcones **6a**–**c** were synthesized using the previously reported procedure [38].

#### 3.1.2. General Procedure for the Synthesis of 2-[4-(E)-3-Arylacryloylphenoxy]-N-[4-(E)-3-arylacryloylphenyl]Acetamides (7a-j)

4-Hydroxychalcones **6a**–**c** (0.02 mol) was added to a solution of acetylated chalcone **4a**–**f** (0.02 mol) and dry K_2_CO_3_ (0.06 mol) dissolved in dry acetone (20 mL) under an inert atmosphere. After stirring for 18–20 h at reflux, the reaction was cooled, and the reaction mixture was concentrated under reduced pressure. The residue was washed using hot distilled water and filtered. The crude residue was purified via crystallization from ethanol to afford bis-chalcone **7a**–**j**.

#### 3.1.3. General Procedure for the Synthesis of Bis-Pyrazoline Hybrid Hits (8–17)

A mixture of bis-chalcone **7a**–**j** (0.01 mol) and hydrazine hydrate (0.08 mol) was heated at reflux for 10 h in absolute ethanol (50 mL). The solution was left to cool at room temperature, then left in a refrigerator overnight, and the solid formed was filtered off using suction, washed with cold ethanol and diethyl ether, and dried under ambient temperature in an inert atmosphere without further crystallization affording bis-pyrazoline novel hybrids **8**–**17** as white pure powder.

2-(4-(5-(4-Chlorophenyl)-4,5-dihydro-1H-pyrazol-3-yl)phenoxy)-N-(4-(5-phenyl-4,5-dihydro-1H-pyrazol-3-yl)phenyl)acetamide (**8**).

Yield 89%, white flakes; mp 263–264°C; ^1^H NMR (500 MHz, DMSO-*d_6_*) δ (ppm): 10.22 (1H, s, O = C-NH), 7.68 (2H, d, *J* = 8.50 Hz, Ar-H), 7.55-7.60 (4H, m, Ar-H), 7.51-7.25 (11H, m, 9Ar-H + 2 pyrazoline NH), 7.04 (2H, d, *J* = 8.50 Hz, Ar-H), 4.85-4.79 (2H, m, pyrazoline H), 4.75 (2H, s, OCH_2_), 3.46-3.40 (2H, m, pyrazoline H), 2.85-2.77 (2H, m, pyrazoline H); ^13^C NMR (125 MHz, DMSO-*d_6_*) δ (ppm): 166.91, 158.37, 149.13, 148.87, 143.53, 142.66, 138.71, 132.02, 129.20, 129.02, 128.86, 128.80, 127.59, 127.39, 127.32, 127.10, 126.95, 126.44, 120.00, 115.22, 115.19, 67.62, 64.11, 63.29, 41.26, 41.15; anal. calcd. For C_32_H_28_ClN_5_O_2_ (550.06): C, 69.87; H, 5.13; N, 12.73. Found: C, 69.75; H, 5.30; N, 12.98.

2-(4-(5-(4-Chlorophenyl)-4,5-dihydro-1H-pyrazol-3-yl)phenoxy)-N-(4-(5-(4-methoxyphenyl)-4,5-dihydro-1H-pyrazol-3-yl)phenyl)acetamide (**9**).

Yield 87%, white fluffy solid; mp 248–249 °C; ^1^H NMR (500 MHz, DMSO-*d_6_*) δ (ppm): 10.20 (1H, s, O = C-NH), 7.68 (2H, d, *J* = 8.60 Hz, Ar-H), 7.60 (4H, d, *J* = 8.60 Hz, Ar-H), 7.42 (1H, s, pyrazoline NH), 7.33-7.27 (5H, m, 4 Ar-H+ pyrazoline NH), 7.03 (2H, d, *J* = 8.50 Hz, Ar-H), 6.91-6.89 (4H, m, Ar-H), 4.79-4.73 (4H, m, 2 pyrazoline H + OCH_2_), 3.74 (3H, s, OCH_3_), 3.39-3.35 (2H, m, pyrazoline H), 2.82-2.76 (2H, m, pyrazoline H); ^13^C NMR (125 MHz, DMSO-*d_6_*) δ (ppm): 166.91, 158.91, 158.30, 149.06, 148.91, 138.67, 135.44, 135.38, 129.30, 128.21, 127.31, 127.19, 126.90, 126.42, 126.39, 120.01, 119.57, 115.22, 114.24, 112.56, 67.67, 63.65, 63.59, 55.55, 41.24, 41.08; anal. calcd. For C_33_H_30_ClN_5_O_3_ (580.08): C, 68.33; H, 5.21; N, 12.07. Found: C, 68.54; H, 5.36; N, 12.31.

2-(4-(5-(4-Chlorophenyl)-4,5-dihydro-1H-pyrazol-3-yl)phenoxy)-N-(4-(5-(4-bromophenyl)-4,5-dihydro-1H-pyrazol-3-yl)phenyl)acetamide (**10**).

Yield 88%, white solid; mp 265–266 °C; ^1^H NMR (500 MHz, DMSO-*d_6_*) δ (ppm): 10.24 (1H, s, O = C-NH), 7.67 (2H, d, *J* = 8.60 Hz, Ar-H), 7.59–7.53 (7H, m, 5 Ar-H+ pyrazoline NH), 7.45 (1H, s, pyrazoline NH), 7.40–7.33 (6H, m, Ar-H), 7.03 (2H, d, *J* = 8.50 Hz, Ar-H), 4.79–4.83 (2H, m, pyrazoline H), 4.74 (2H, s, OCH_2_), 3.50–3.40 (2H, m, pyrazoline H), 2.76–2.82 (2H, m, pyrazoline H); ^13^C NMR (125 MHz, DMSO-*d_6_*) δ (ppm): 166.95, 158.35, 149.18, 149.03, 143.07, 143.01, 142.62, 138.76, 132.02, 131.73, 129.40, 129.02, 128.80, 127.41, 126.91, 126.49, 120.53, 120.00, 115.21, 67.56, 63.36, 63.26, 41.25, 41.06; anal. calcd. For C_32_H_27_BrClN_5_O_2_ (628.95): C, 61.11; H, 4.33; N, 11.14; found: C, 61.37; H, 4.50; N, 11.39. 

2-(4-(5-(4-Chlorophenyl)-4,5-dihydro-1H-pyrazol-3-yl)phenoxy)-N-(4-(5-(4-chlorophenyl)-4,5-dihydro-1H-pyrazol-3-yl)phenyl)acetamide (**11**).

Yield 81%, white solid; mp 273–274 °C; ^1^H NMR (500 MHz, DMSO-*d_6_*) δ (ppm): 10.21 (1H, s, O = C-NH), 7.68 (2H, d, *J* = 8.67 Hz, Ar-H), 7.60–7.58 (4H, m, Ar-H), 7.45–7.40 (6H, m, 4Ar-H+ 2 pyrazoline NH), 7.27 (2H, d, *J* = 8.60 Hz, Ar-H), 7.16 (2H, d, *J* = 8.50 Hz, Ar-H), 7.03 (2H, d, *J* = 8.05 Hz, Ar-H), 4.84–4.76 (2H, m, pyrazoline H), 4.74 (2H, s, OCH_2_), 3.47–3.41 (2H, m, pyrazoline H), 2.83–2.76 (2H, m, pyrazoline H); ^13^C NMR (125 MHz, DMSO-*d_6_*) δ (ppm): 166.89, 158.38, 149.10, 148.84, 142.67, 140.52, 138.68, 136.64, 132.02, 129.39, 129.02, 128.80, 127.38, 127.32, 126.98, 126.47, 126.40, 120.00, 115.23, 67.65, 63.87, 63.28, 41.26, 41.08; anal. calcd. For C_32_H_27_Cl_2_N_5_O_2_ (584.50): C, 65.76; H, 4.66; N, 11.98; found: C, 65.91; H, 4.73; N, 12.15. 

2-(4-(5-(4-Methoxyphenyl)-4,5-dihydro-1H-pyrazol-3-yl)phenoxy)-N-(4-(5-(3,4-dimethoxyphenyl)-4,5-dihydro-1H-pyrazol-3-yl)phenyl)acetamide (**12**).

Yield 85%, white flakes; mp 279–280 °C; ^1^H NMR (500 MHz, DMSO-*d_6_*) δ (ppm): 10.22 (1H, s, O = C-NH), 7.68 (2H, d, *J* = 8.68 Hz, Ar-H), 7.60 (4H, m, Ar-H), 7.43 (1H, s, pyrazoline NH), 7.33–7.28 (3H, m, 2Ar-H + pyrazoline NH), 7.03–7.00 (3H, m, Ar-H), 6.91-6.89 (4H, m, Ar-H), 4.79–4.73 (4H, m, 2 pyrazoline H + OCH_2_), 3.75–3.73 (9H, m, 3 OCH_3_), 3.40–3.34 (2H, m, pyrazoline H), 2.84–2.75 (2H, m, pyrazoline H); ^13^C NMR (125 MHz, DMSO-*d_6_*) δ (ppm): 166.92, 158.88, 158.28, 149.15, 149.05, 149.03, 148.43, 138.68, 135.77, 135.43, 129.29, 128.21, 127.31, 127.18, 126.40, 119.99, 119.10, 115.20, 114.22, 112.16, 110.86, 67.64, 64.03, 63.60, 56.03, 55.89, 55.53, 41.24, 41.11; anal. calcd. For C_35_H_35_N_5_O_5_ (605.69): C, 69.41; H, 5.82; N, 11.56; found C, 69.29; H, 5.98; N, 11.75. 

2-(4-(5-(4-Methoxyphenyl)-4,5-dihydro-1H-pyrazol-3-yl)phenoxy)-N-(4-(5-(2,4-dimethylphenyl)-4,5-dihydro-1H-pyrazol-3-yl)phenyl)acetamide (**13**).

Yield 86%, white flakes; mp 244–245 °C; ^1^H NMR (500 MHz, DMSO-*d_6_*) δ (ppm): 10.21 (1H, s, O = C-NH), 8.17–8.14 (1H, m, Ar-H), 7.99–7.96 (1H, m, Ar-H), 7.86–7.78 (2H, m, Ar-H + pyrazoline NH), 7.67 (1H, d, *J* = 8.66 Hz, Ar-H), 7.60–7.57 (3H, m, 2Ar-H + pyrazoline NH), 7.31 (2H, d, *J* = 8.55 Hz, Ar-H), 7.18–7.15 (1H, m, Ar-H), 7.11 (1H, d, *J* = 8.50 Hz, Ar-H), 7.03–6.96 (4H, m, Ar-H), 6.91 (1H, d, *J* = 8.60 Hz, Ar-H), 4.97–4.90 (2H, m, pyrazoline H), 4.74 (2H, s, OCH_2_), 3.74 (3H, s, OCH_3_), 3.48–3.43 (2H, m, pyrazoline H), 2.78 (1H, dd, *J* = 16.20, *J*  = 6.50 Hz, pyrazoline H), 2.66 (1H, dd, *J* = 16.20, *J*  = 6.50 Hz, pyrazoline H), 2.30 (3H, s, CH3), 2.24 (3H, s, CH3); ^13^C NMR (125 MHz, DMSO-*d_6_*_)_ δ (ppm): 166.90, 158.90, 149.05, 148.44, 138.54, 136.17, 135.44, 135.26, 132.67, 131.38, 129.91, 129.34, 128.20, 127.31, 126.92, 126.37, 126.07, 120.00, 115.22, 114.64, 114.24, 67.67, 63.59, 60.75, 55.55, 41.24, 40.50, 21.01, 19.44; anal. calcd. For C_35_H_35_N_5_O_3_ (573.70): C, 73.28; H, 6.15; N, 12.21; found: C, 73.12; H, 6.29; N, 12.47.

2-(4-(5-(4-Methoxyphenyl)-4,5-dihydro-1H-pyrazol-3-yl)phenoxy)-N-(4-(5-(4-bromophenyl)-4,5-dihydro-1H-pyrazol-3-yl)phenyl)acetamide (**14**).

Yield 83%, white fluffy solid; mp 283–284 °C; ^1^H NMR (500 MHz, DMSO-*d_6_*) δ (ppm): 10.22 (1H, s, O = C-NH), 7.67 (2H, d, *J* = 8.60 Hz, Ar-H), 7.59–7.53 (5H, m, 4Ar-H + pyrazoline NH), 7.41–7.28 (7H, m, 6Ar-H + pyrazoline NH), 7.02 (2H, d, *J* = 8.50 Hz, Ar-H), 6.90 (2H, d, *J* = 8.60 Hz, Ar-H), 4.86–4.74 (4H, m, 2 pyrazoline H + OCH_2_), 3.73 (3H, s, OCH_3_), 3.47–3.34 (2H, m, pyrazoline H), 2.83–2.75 (2H, m, pyrazoline H); ^13^C NMR (125 MHz, DMSO-*d_6_*) δ (ppm): 166.94, 158.88, 158.28, 149.07, 148.99, 142.60, 138.78, 135.42, 132.04, 129.05, 129.02, 128.81, 128.21, 127.31, 127.17, 126.48, 120.00, 115.20, 114.22, 67.62, 63.59, 63.34, 55.53, 41.23, 41.10; anal. calcd. For C_33_H_30_BrN_5_O_3_ (624.54): C, 63.46; H, 4.84; N, 11.21; found: C, 63.70; H, 4.95; N, 11.43. 

2-(4-(5-(4-Methoxyphenyl)-4,5-dihydro-1H-pyrazol-3-yl)phenoxy)-N-(4-(5-(4-chlorophenyl)-4,5-dihydro-1H-pyrazol-3-yl)phenyl)acetamide (**15**).

Yield 89%, white fluffy solid; mp 273–274 °C; ^1^H NMR (500 MHz, DMSO-*d_6_*) δ (ppm): 10.22 (1H, s, O = C-NH), 7.87–7.58 (9H, m, 7Ar-H + 2 pyrazoline NH), 7.30–6.89 (9H, m, Ar-H), 4.87–4.81 (2H, m, pyrazoline H), 4.75 (2H, s, OCH_2_), 3.73 (3H, s, OCH_3_), 3.47–3.41 (2H, m, pyrazoline H), 2.83–2.75 (2H, m, pyrazoline H); ^13^C NMR (125 MHz, DMSO-*d_6_*) δ (ppm): 166.93, 158.88, 158.29, 148.97, 142.60, 138.80, 135.39, 132.04, 129.03, 128.81, 128.22, 127.32, 126.88, 126.48, 119.98, 115.30, 115.20, 114.63, 114.22, 67.62, 63.60, 63.35, 55.53, 41.24, 41.11; anal. calcd. For C_33_H_30_ClN_5_O_3_ (580.08): C, 68.33; H, 5.21; N, 12.0; found: C, 68.57; H, 5.34; N, 12.29. 

2-(4-(5-(4-Methoxyphenyl)-4,5-dihydro-1H-pyrazol-3-yl)phenoxy)-N-(4-(5-(4-fluorophenyl)-4,5-dihydro-1H-pyrazol-3-yl)phenyl)acetamide (**16**).

Yield 84%, white solid; mp 252–253°C; ^1^H NMR (500 MHz, DMSO-*d_6_*) δ (ppm): 10.20 (1H, s, O = C-NH), 7.68–7.58 (6H, m, Ar-H), 7.45 (1H, s, pyrazoline NH), 7.31–7.29 (5H, m, 4Ar-H + pyrazoline NH), 7.01 (2H, d, *J* = 8.60 Hz, Ar-H), 6.91–6.89 (4H, m, Ar-H), 4.79–4.73 (4H, m, 2 pyrazoline H + OCH_2_), 3.74 (3H, s, OCH_3_), 3.39–3.35 (2H, m, pyrazoline H), 2.82–2.76 (2H, m, pyrazoline H); ^13^C NMR (125 MHz, DMSO-*d_6_*) δ (ppm): 166.92, 163.55, 158.90, 148.99, 129.98, 129.91, 129.07, 129.01, 128.21, 127.60, 127.32, 126.89, 126.45, 120.01, 115.64, 115.47, 115.23, 114.64, 114.24, 67.68, 63.59, 63.39, 55.55, 41.24, 41.16; anal. calcd. For C_33_H_30_FN_5_O_3_ (563.63): C, 70.32; H, 5.37; N, 12.43; found: C, 70.54; H, 5.51; N, 12.69.

2-(4-(5-(3,4-Dimethoxyphenyl)-4,5-dihydro-1H-pyrazol-3-yl)phenoxy)-N-(4-(5-(4-methoxyphenyl)-4,5-dihydro-1H-pyrazol-3-yl)phenyl)acetamide (**17**).

Yield 88%, white fluffy solid; mp 281–282 °C; ^1^H NMR (500 MHz, DMSO-*d_6_*) δ (ppm): 10.20 (1H, s, O = C-NH), 8.18–8.14 (2H, m, Ar-H), 7.84 (2H, s, pyrazoline NH), 7.67 (2H, d, *J* = 8.50 Hz, Ar-H), 7.59–7.61 (3H, m, 2Ar-H + pyrazoline NH), 7.30 (1H, d, *J* = 8.50 Hz, Ar-H), 7.13–7.09 (2H, m, Ar-H), 7.05–7.00 (3H, m, Ar-H), 6.91–6.89 (3H, m, Ar-H), 4.80–4.74 (4H, m, 2 pyrazoline H + OCH_2_), 3.75–3.73 (9H, m, 3 OCH_3_), 3.39–3.43 (2H, m, pyrazoline H), 2.85–2.77 (2H, m, pyrazoline H); ^13^C NMR (125 MHz, DMSO-*d_6_*) δ (ppm): 166.91, 158.92, 158.34, 149.19, 148.48, 138.71, 135.74, 135.34, 129.98, 128.23, 127.35, 127.14, 126.89, 126.42, 120.01, 119.13, 115.23, 114.64, 114.24, 112.24, 110.95, 67.68, 64.01, 63.57, 56.07, 55.93, 55.55, 41.24, 41.11; anal. calcd. For C_35_H_35_N_5_O_5_ (605.69): C, 69.41; H, 5.82; N, 11.56; found: C, 69.67; H, 5.94; N, 11.81.

### 3.2. Biology

#### 3.2.1. Cell Viability Assay

The normal human mammary gland epithelial (MCF-10A) cell line was used to test the viability of new compounds [46,47]. See Appendix SA (Supplementary File).

#### 3.2.2. Antiproliferative Assay

The antiproliferative activity of pyrazoline hybrids 8–17 was tested against the four human cancer cell lines Panc-1 (pancreatic cancer cell line), MCF-7 (breast cancer cell line), HT-29 (colon cancer cell line), and A-549 (lung cancer cell line) using the MTT assay and doxorubicin as the reference drug [48,49,50]. See Appendix SA (Supplementary File).

#### 3.2.3. EGFR Inhibitory Assay

Compounds **12**, **14**, **15**, and **17** were tested for EGFR inhibitory activity as a potential target for their antiproliferative activity [51]. See Appendix SA (Supplementary File).

#### 3.2.4. BRAF^V600E^ Inhibitory Assay

The antiBRAF^V600E^ activity of compounds **12**, **14**, **15**, and **17** was also investigated in vitro [51] using erlotinib as a reference compound. See Appendix SA (Supplementary File).

#### 3.2.5. Apoptotic Marker Assay

##### Caspase 3 Activation Assay

Compounds **12** and **17** were tested as caspase-3 activators against the human pancreatic (Panc-1) cancer cell line [47]. See Appendix SA (Supplementary File).

##### Caspase-8, Bax, and Bcl-2 Level Assays

Compounds **12** and **17** were further investigated for their impact on caspase-8, Bax, and Bacl-2 levels against the Panc-1 cancer cell line and staurosporine was used as a reference [53]. See Appendix SA (Supplementary File).

### 3.3. Statistical Analysis

The statistical analysis of the data was conducted using Prism 9 software. The data were analyzed using a one-way ANOVA test followed by Tukey’s post-ANOVA test for multiple comparisons at a significance level of *p* ≤ 0.05. The results were presented as mean ± standard error of the mean (SEM).

### 3.4. Docking Study

In a molecular docking study, BIOVIA I Discovery Studio 2016 software was used. The selected proteins were prepared for docking studies using the Protein Preparation Wizard. The ligands were mapped onto a three-dimensional model and subjected to energy minimization using LigPrep. To optimize the potential binding, a receptor grid was generated for the selected binding site using the Receptor Grid Generation Tool. Finally, the Glide tool was used to evaluate the docking score and different binding modes for the ligands.

### 3.5. Quantum Chemical Calculations

The Gaussian 09 program was used to perform structural optimization and DFT calculations. Becke’s B3LYP exchange correlation functional and the 6-311 G (d,p) basis set were used to determine the energy minima. Solvent effects were not adjusted for, and GaussView 6.0 was used for the visualization and plots.

### 3.6. In Silico ADMET Analysis

BIOVIA I Discovery Studio 2016 was used to conduct the ADMET studies. The chemical structures of all of the compounds were imported and ADMET descriptors were predicted using built-in models such as Lipinski’s rule of five, absorption, distribution, metabolism, excretion, and toxicity. The results were analyzed to determine the drug-likeness and safety of compounds.

## 4. Conclusions

With the goal of developing a new dual-targeting antiproliferative agent, a new series of bis-pyrazoline derivatives was designed and synthesized. The MTT assay was used to test new compounds’ antiproliferative effects against a panel of four cancer cell lines. Compounds **12**, **15**, and **17** had the highest antiproliferative activity, with GI_50_ values of 1.05, 1.50, and 1.20 µM, respectively, when compared to doxorubicin, which had a GI_50_ of 1.10 µM. The most potent compounds were then tested for the inhibition of EGFR and BRAF^V600E^. The results showed that compounds **12**, **15**, and **17** were the most potent EGFR inhibitors, with IC_50_ values of 81, 93, and 87 nM, respectively. Furthermore, **12**, **15**, and **17** showed promising BRAF^V600E^ inhibitory activity. As a result, they may be effective antiproliferative agents that function as dual EGFR/BRAF^V600E^ inhibitors. Furthermore, caspase 3 overexpression and an increased ratio of Bax/Bcl-2 genes in MCF-7 cells may be responsible for the induction of apoptosis for the most active derivatives: **12** and **17**. In silico molecular docking studies discovered that compound **12** was a potent inhibitor of both EGFR and BRAF^V600E^ targets. According to the ADMET study, the majority of the compounds have little toxicity or unfavorable side effects on the tested models. Hybrid **12** is a suitable ligand for EGFR and BRAF^V600E^ because it is softer than hybrid **15** according to the DFT computational data and electrostatic potential maps. More in vitro and in vivo studies, as well as chemical modifications, may be required in the future to develop highly effective antiproliferative agents.

## Figures and Tables

**Table 1:** IC_50_ of compounds **8**–**17** and Erlotinib.

Comp.	Cell Viability %	Antiproliferative Activity IC_50_ ± SEM (µM)				
		A-549	MCF-7	Panc-1	HT-29	Average IC_50_ (GI_50_)
**8**	90	4.30 ± 0.40	4.10 ± 0.40	4.60 ± 0.40	4.50 ± 0.40	4.40
**9**	89	2.20 ± 0.20	1.90 ± 0.10	2.60 ± 0.20	2.50 ± 0.20	2.30
**10**	89	3.20 ± 0.30	2.90 ± 0.20	3.20 ± 0.30	3.10 ± 0.30	3.10
**11**	87	2.90 ± 0.20	2.70 ± 0.20	2.90 ± 0.20	3.00 ± 0.20	2.90
**12**	90	1.00 ± 0.10	0.90 ± 0.10	1.10 ± 0.10	1.20 ± 0.10	1.05
**13**	87	4.80 ± 0.40	4.60 ± 0.40	4.90 ± 0.40	5.00 ± 0.40	4.80
**14**	89	1.80 ± 0.10	1.60 ± 0.10	2.00 ± 0.10	2.00 ± 0.10	1.85
**15**	90	1.40 ± 0.10	1.20 ± 0.10	1.70 ± 0.10	1.70 ± 0.10	1.50
**16**	89	3.70 ± 0.30	3.50 ± 0.30	3.80 ± 0.30	3.90 ± 0.30	3.70
**17**	91	1.20 ± 0.10	1.00 ± 0.10	1.30 ± 0.10	1.30 ± 0.10	1.20
Doxorubicin	--	1.40 ± 0.08	0.90 ± 0.02	1.00 ± 0.02	1.20 ± 0.03	1.10

**Table 2:** IC_50_ of compounds **12**, **14**, **15**, and **17** against EGFR.

Compound	EGFR Inhibition IC_50_ ± SEM (nM)	BRAF^V600E^ Inhibition IC_50_ ± SEM (nM)
**12**	81 ± 5	93 ± 6
**14**	104 ± 9	125 ± 10
**15**	93 ± 7	98 ± 8
**17**	87 ± 6	107 ± 9
Erlotinib	80 ± 5	60 ± 5

**Table 3:** Caspase-3 induction of compounds **12** and **17**.

Compound Number	Caspase–3	
	Conc (pg/mL)	Fold Change
**12**	543.50 ± 4	8.5
**17**	469.50 ± 4	7.2
Staurosporine	503.00 ± 4	8.0
Control	65.50	1

**Table 4:** Caspase-8, Bax, and Bcl-2 levels for **12**, **17**, and staurosporine.

Compound Number	Caspase-8		Bax		Bcl-2	
	Conc (ng/mL)	Fold Change	Conc (pg/mL)	Fold Change	Conc (ng/mL)	Fold Reduction
**12**	1.87	21	296	37	1.05	5
**17**	1.77	20	285	35	1.15	4
Staurosporine	1.80	20	280	35	1.10	5
Control	0.09	1	8	1	5	1

**Table 5:** Quantum global reactivity parameters for hybrid **12** and **15** bis pyrazolines which were calculated via the DFT method using B3LYP/6-31G (d,p).

Comp. ID	12	15
E_HOMO_ (eV)	−5.127	−5.231
E_LUMO_ (eV)	−1.165	−1.213
E_LUMO_-E_HOMO_ (eV)	3.962	4.018
Ionization potential (IP)	5.127	5.231
Electron affinity (EA)	1.165	1.213
Electronegativity (χ)	3.146	3.222
Chemical potential (μ)	−3.146	−3.222
Chemical hardness (η)	1.981	2.009
Chemical softness (S)	0.252	0.249

**Table 6:** Predicted ADMET for synthesized hybrids.

Comp. ID	PSA	PPB ^a^	Absorption Level ^b^	CYP2D6 Prediction ^c^	BBB Level ^d^	Solubility Level ^e^	AlogP98
**8**	87.308	TRUE	2	FALSE	4	1	6
**9**	96.438	TRUE	2	FALSE	4	2	5.984
**10**	87.515	TRUE	2	FALSE	4	1	6.749
**11**	88.308	TRUE	2	FALSE	4	1	6.665
**12**	116.098	FALSE	2	FALSE	4	2	5.287
**13**	99.738	FALSE	2	FALSE	4	2	6.292
**14**	98.938	FALSE	2	FALSE	4	2	6.068
**15**	95.648	TRUE	2	FALSE	4	2	5.984
**16**	97.579	TRUE	2	FALSE	4	2	5.525
**17**	114.098	FALSE	2	FALSE	4	2	5.287
Erlotinib	71.052	TRUE	0	FALSE	1	2	4.309

^a^ PBB, plasma protein binding; FALSE means less than 90%; TRUE means > 90%. ^b^ Absorption level, 0 = good, 1 = moderate, 2 = poor, 3 = very poor. ^c^ CYP2D6, cytochrome P2D6; TRUE = inhibitor; FALSE = non-inhibitor. ^d^ BBB level, blood–brain barrier level, 0 = very high, 1 = high, 2 = medium, 3 = low, 4 = very low. ^e^ Solubility level, 1 = very low, 2 = low, 3 = good, 4 = optimal.

**Table 7:** Predicted toxicity properties of synthesized hybrids.

Comp. ID	FDA Rodent Carcinogenicity (Mouse–Female)	Rat Maximum Tolerated Dose (Feed) ^a^	Rat Oral LD50 ^a^	Ocular Irritancy	Skin Irritancy
**8**	Non-Carcinogenic	0.271	0.871442	Moderate	Non-Irritant
**9**	Non-Carcinogenic	0.1722	1.35834	Mild	Non-Irritant
**10**	Non-Carcinogenic	0.1377	1.39385	Moderate	Non-Irritant
**11**	Non-Carcinogenic	0.2145	1.15043	Moderate	Non-Irritant
**12**	Non-Carcinogenic	0.1287	10.2483	Mild	Non-Irritant
**13**	Non-Carcinogenic	0.1108	2.4424	Mild	Non-Irritant
**14**	Non-Carcinogenic	0.1106	1.80353	Mild	Non-Irritant
**15**	Non-Carcinogenic	0.1722	1.35834	Mild	Non-Irritant
**16**	Non-Carcinogenic	0.1878	0.707581	Mild	Non-Irritant
**17**	Non-Carcinogenic	0.1287	10.2483	Mild	Non-Irritant
Erlotinib	Non-Carcinogenic	0.0828	0.662169	Mild	Non-Irritant

^a^ Unit: g/kg body weight.

## Data Availability

The data will be provided upon request.

## Supplementary Materials

The following supporting information can be downloaded at https://www.mdpi.com/article/10.3390/ijms24109104/s1.
